# Supplementary material for: First Assessment of Micro-Litter Ingested by Dolphins, Sea Turtles and Monk Seals Found Stranded along the Coasts of Samos Island, Greece
Source: Animals (Basel). 2022 Dec 11;12(24):3499. doi: 10.3390/ani12243499 (PMC9774117; doi:10.3390/ani12243499)
Supplement: Supplementary file 1 [file animals-12-03499-s001.zip › animals-2072589-supplementary.pdf]

Supplementary materials to

First assessment of micro-litter ingested by dolphins, sea turtles and monk seals found stranded along the coasts of Samos Is-land, Greece

Guido Pietroluongo[1,2*], Belén Quintana Martín-Montalvo[1], Simone Antichi[1,3], Anastasia Miliou[1], Valentina Costa[1,4*]

1 Archipelagos Institute of Marine Conservation, P.O. Box 42, 83103 Pythagorio, Samos, Greece
2 Department of Comparative Biomedicine and Food Science, University of Padova, Viale dell'Università 16, 35020 Legnaro, Italy
3 Departamento de Ciencias Marinas y Costeras, Universidad Autónoma de Baja California Sur, Sur KM 5.5, La Paz 23080, México
4 Stazione Zoologica Anton Dohrn (SZN), Department of Integrative Marine Ecology, Contrada Torre Spaccata, Località Torre Spaccata, 87071 Amendolara, Italy
* Correspondence: guido.pietroluongo@gmail.com (G.P.); valentina.costa@szn.it (V.C.)

Table S1. Number of MPs detected in the laboratory (mean ± Standard Deviation, S.D.) and assessed on GFF filters (n = 10) before (pre-test) and during the manipulation (test).

Test	Type	Size (mm)	Color	Number of MPs Mean	S.D.
pre-test	fibre	0.20-0.50	black	1	0
			pink	1	0
		1.01-2.50	clear	1	0
			yellow	1	0
	fragment	0.20-0.50	white	1	0
test	fibre	0.20-0.50	black	1	0
			blue	1	0
			red	2	0
			yellow	1.25	0.5
		0.51-1.00	black	1	0
			blue	1	0
			red	1	0
		1.01-2.50	black	1	0
			blue	1	0
			red	1	0
		2.51-5.00	black	1	0
			blue	1	0
	fragment	0.20-0.50	black	1	0
			blue	1	0
			green	1	0
			yellow	1	0

Table S2. Summary of the most recent studies investigating microplastics (MPs) contamination in the gastrointestinal tracts (GIT) of stranded marine megafauna.

Reference	Family	Species	N. of individuals analysed (with MPs)	Study location	Study date	Method of Quantification	Localization	Tot n. of particles	Particles type	Particle Size range (in mm)
(Bravo Rebolledo et al., 2013)	Phocidae	*Phoca vitulina*	207 (13)	Texel, Netherlands	2002	Visual identification	stomach	28		not stated
							intestines	7		not stated
(Besseling et al., 2015)	Balaenopteridae	*Megaptera novaeangliae*	1 (1)	Texel, Netherlands	2012	Visual identification and FT-IR	GIT	45		0.01-170
(Lusher et al., 2015)	Ziphidae	*Mesoplodon mirus*	3 (3)	Galway, Ireland	2013	Visual identification and FT-IR	GIT (oesophagus, stomachs and intestine)	88	Fibres (n = 69)	0.3-7 (mean 2.16±1.39)
									Fragments (n = 19)	
(Ryan et al., 2016)	Cheloniidae	*Caretta caretta* (post-hatchling)	230 (16)	Western Cape, South Africa	2015	Visual identification	GIT (stomach, intestine, cloaca and bladder)	301	Fragments (n = 229)	ND-17 (mean (4.7±2.4 × 3.0±1.4 × 1.0±0.5)
									Bags (n = 33)	
									Fibres (n =24)	ND-30 (mean 16.3±7.6)
									Pellets (n = 10)	
									Other (n = 5)	
(Pham et al., 2017)	Cheloniidae	*Caretta caretta*	12 stranded (8)	Azores region, Portugal	1996-2016	Visual identification and FT-IR	GIT (oesophagus, stomachs and intestine)	380	Fragments (n = 213)	ND-310 (mean 20.3±1.6)
									Sheets (n = 98)	
									Foam (n = 4)	

									Synthetic rope (n = 38)	
									Fishing line (n = 22)	
									Pellets (n = 5)	
(van Franeker et al., 2018)	Phocoenidae	*Phocoena phocoena*	654 (44)	Texel, Netherlands	2003-2013	Visual identification and NIR	stomach	71		not stated
(Hernandez-Gonzalez et al., 2018)	Delphinidae	*Delphinus delphis*	35 (35)	Gacilian coast, Spain	not stated	Visual identification	stomach(s)	411	Fibres	0.29-4.92 (mean 2.11±1.26)
									Fragments	0.49-4.07 (mean 1.29±0.93)
									Beads	0.95 (diameter)
(Lusher et al., 2018)		NID Mysticeti	45 (0)	Ireland	1990-2015	Visual identification	GIT (oesophagus, stomachs and intestine)	500	Fibres	0.6-16.7
	Balaenopteridae	NID Balaenopteridae	2 (0)							
	Balaenopteridae	*Balaenoptera acutorostrata*	90 (0)							
	Balaenopteridae	*Balaenoptera borealis*	1 (0)							
	Balaenopteridae	*Balaenoptera physalus*	24 (0)							
	Balaenopteridae	*Megaptera novaeangliae*	5 (0)							
	Physeteridae	*Physeter macrocephalus*	57 (0)							
	Physeteridae	*Kogia breviceps*	9 (0)							
		NID Cetacean	50 (0)							
		NID Odontoceti	17 (0)							
		NID beaked whale	10 (0)							

	Ziphidae	*Hyperoodon ampullatus*	16 (0)							
	Ziphidae	*Mesoplodon bidens*	11 (0)							
	Ziphidae	*Mesoplodon mirus*	5 (0)					98	Fragments	0.6-16.7
	Ziphidae	*Ziphius cavirostris*	38 (1)							
	Delphinidae	NID Delphinidae	176 (0)							
	Delphinidae	*Delphinus delphis*	755 (10)							
	Delphinidae	*Stenella coeruleoalba*	219 (2)							
	Delphinidae	Delphinids	17 (0)							
		Delphinidae/Phocoenidae	41 (0)							
	Phocoenidae	*Phocoena phocoena*	608 (5)							
	Delphinidae	*Globicephala melas*	338 (0)							
	Delphinidae	*Grampus griseus*	68 (0)							
	Delphinidae	*Lagenorhynchus acutus*	152 (0)							
	Delphinidae	*Lagenorhynchus albirostris*	25 (0)							
	Delphinidae	*Orcinus orca*	5 (1)							
	Delphinidae	*Tursiops truncatus*	150 (2)							
(Nelms et al., 2019)	Delphinidae	*Lagenorhynchus acutus*	1 (1)	British coast	2014-2017	Visual identification and FT-IR	GIT (stomachs and intestine)	8	Fibres (n = 229)	0.10-200 (mean 2±2.3)
	Delphinidae	*Tursiops truncatus*	1 (1)					6		
	Delphinidae	*Delphinus delphis*	16 (16)					91		
	Phocidae	*Halichoerus grypus*	3 (3)					18		
	Phocoenidae	*Phocoena phocoena*	21 (21)					110		
	Phocidae	*Phoca vitulina*	4 (4)					17	Fragments (n = 44)	0.1-4 (mean 0.9±1.1)
	Physeteridae	*Kogia breviceps*	1 (1)					4		
	Delphinidae	*Grampus griseus*	1 (1)					9		
	Delphinidae	*Stenella coeruleoalba*	1 (1)					7		
	Delphinidae	*Lagenorhynchus albirostris*	1 (1)					3		

(Duncan et al., 2019)	Cheloniidae	*Chelonia mydas*	34 (34)	Cyprus Island, Greece	2011-16	Visual identification and FT-IR	GIT (entire)	12	Fibres	Mediterranean: ND-1.40±0.54; Atlantic: ND-2.87±0.20; Pacific: ND-2.85±0.23
	Cheloniidae	*Caretta caretta*	22 (22)	Cyprus Island, Greece	2011-16			13		
	Cheloniidae	*Chelonia mydas*	10 (10)	North Carolina, USA	2016-17			4		
	Cheloniidae	*Caretta caretta*	8 (8)	North Carolina, USA	2016-17			1		
	Cheloniidae	*Lepidochelys kempii*	10 (10)	North Carolina, USA	2010-17			1		
	Dermochelyidae	*Dermochelys coriacea*	2 (2)	North Carolina, USA	2017			1	Fragments and bead	Mediterranean: ND-0.07±0.01; Atlantic: ND-0.31±0.04; Pacific: ND-0.26±0.01
	Cheloniidae	*Chelonia mydas*	7 (7)	Queensland, Australia	1993-2017			4		
	Cheloniidae	*Caretta caretta*	3 (3)	Queensland, Australia	2009-14			2		
	Cheloniidae	*Natator depressus*	4 (4)	Queensland, Australia	2006-14			2		
	Cheloniidae	*Lepidochelys olivacea*	1 (1)	Queensland, Australia	2016			0		
	Cheloniidae	*Eretmochelys imbricata*	1 (1)	Queensland, Australia	2016			0		
(Zhu et al., 2019)	Delphinidae	*Sousa chinensis*	3 (3)	Guangxi Beibu Gulf, China	2015	Visual identification and FT-IR	Foregut (random samples)	45	Fibres, fragments and flakes	0.1-4.8 (mean 2.2±0.4)

							Midgut (random samples)	30		
							Mix-gut (random samples)	2		
(Battaglia et al., 2020)	Delphinidae	*Tursiops truncatus*	7 (7)	South Carolina, USA	2017-2018	Visual identification and FT-IR	GIT (stomachs and intestine)	1964	Fibres (n = 1495)	0.125-5.00
									Fragments (n = 103)	
									Film (n = 336)	
									Foam (N = 30)	
(Digka et al., 2020)	Cheloniidae	*Caretta caretta*	28 stranded (26)	Greece	2017-2019	Visual identification and FT-IR	GIT (oesophagus, stomach, intestine)	286	Threads (n = 128)	0.4-550 (mean 47±3.6)
									Sheets (n = 103	
									Fragments (n = 46)	
									Foam (n = 6)	
									NC (n = 3)	
(Novillo et al., 2020)	Delphinidae	*Stenella coeruleoalba*	43 (39)	Valencian Community, Spain	1988-2017	Visual identification and FT-IR	Digestive tract	672	Fibres (n = 494)	-
									Fragments (n = 160)	-
									Pellet (n = 17)	-
(Zhang et al., 2021)	Delphinidae	*Sousa chinensis*	12 (12)	Pearl river Estuary, China	2016-2018	Visual identification and FT-IR	Stomach	635	Fibres (n = 443)	1.69±1.04
									Film (n = 30)	-
									Pellet (n = 19)	Ø 0.72 (SD = 0.24)
									Irregular particle (n = 143)	0.78±0.71

(Philipp et al., 2021)	Phocoenidae	*Phocoena phocoena*	30 (28)	Schleswig-Holstein, Germany	2014-2018	Visual identification and FT-IR	GIT (intestine)	401	Fibres (n = 202)	0.1-2.0
									Fragments (n = 199)	-
**This study**	Cheloniidae	*Caretta caretta*	9 (9)	Samos Island, Greece	2018-2019	Visual identification	GIT (oesophagus, stomach, intestine)	1969	Fibres (n = 8560)	0.21-5.00 (0.21-0.50, n = 4634; 0.51-1.00, n = 2061; 1.01-2.50 n = 2174; ≤0.20, n = 701; 2.51-5.00, n = 1069)¶
	Cheloniidae	*Chelonia mydas*	6 (6)					1944		
	Phocidae	*Monachus monachus*	2 (2)					538		
	Delphinidae	*Delphinus delphis*	3 (3)					1442	Fragments (n = 2079)	
	Delphinidae	*Stenella coeruleoalba*	3 (3)					3116		
	Delphinidae	*Tursiops truncatus*	2 (2)					1630		

NID: Not Identified; NC: Not Classified

References

Battaglia, F.M., Beckingham, B.A., McFee, W.E., 2020. First report from North America of microplastics in the gastrointestinal tract of stranded bottlenose dolphins (Tursiops truncatus). Mar. Pollut. Bull. 160, 111677. https://doi.org/10.1016/j.marpolbul.2020.111677

Besseling, E., Foekema, E.M., Franeker, J.A. Van, Leopold, M.F., Kühn, S., Rebolledo, E.L.B., Heße, E., Mielke, L., Ijzer, J., Kamminga, P., Koelmans, A.A., 2015. Microplastic in a macro filter feeder : Humpback whale Megaptera novaeangliae. Mar. Pollut. Bull. 95, 248–252. https://doi.org/10.1016/j.marpolbul.2015.04.007

Bravo Rebolledo, E.L., Van Franeker, J.A., Jansen, O.E., Brasseur, S.M., 2013. Plastic ingestion by harbour seals (Phoca vitulina) in The Netherlands. Mar. Pollut. Bull. 67, 200–202. https://doi.org/10.1016/j.marpolbul.2012.11.035

Digka, N., Bray, L., Tsangaris, C., Andreanidou, K., Kasimati, E., Kofidou, E., Komnenou, A., Kaberi, H., 2020. Evidence of ingested plastics in stranded loggerhead sea turtles along the Greek coastline, East Mediterranean Sea. Environ. Pollut. 263. https://doi.org/10.1016/j.envpol.2020.114596

Duncan, E.M., Broderick, A.C., Fuller, W.J., Galloway, T.S., Godfrey, M.H., Hamann, M., Limpus, C.J., Lindeque, P.K., Mayes, A.G., Omeyer, L.C.M., Santillo, D., Snape, R.T.E., Godley, B.J., 2019. Microplastic ingestion ubiquitous in marine turtles. Glob. Chang. Biol. 25, 744–752. https://doi.org/10.1111/gcb.14519

Hernandez-Gonzalez, A., Saavedra, C., Gago, J., Covelo, P., Santos, M.B., Pierce, G.J., 2018. Microplastics in the stomach contents of common dolphin (Delphinus delphis) stranded on the Galician coasts (NW Spain, 2005–2010). Mar. Pollut. Bull. 137, 526–532. https://doi.org/10.1016/j.marpolbul.2018.10.026

Lusher, A.L., Hernandez-Milian, G., Berrow, S., Rogan, E., O'Connor, I., 2018. Incidence of marine debris in cetaceans stranded and bycaught in Ireland: Recent findings and a review of historical knowledge. Environ. Pollut. 232, 467–476. https://doi.org/10.1016/j.envpol.2017.09.070

Lusher, A.L., Hernandez-Milian, G., O'Brien, J., Berrow, S., O'Connor, I., Officer, R., 2015. Microplastic and macroplastic ingestion by a deep diving, oceanic cetacean: The True's beaked whale Mesoplodon mirus. Environ. Pollut. 199, 185–191. https://doi.org/10.1016/j.envpol.2015.01.023

Nelms, S.E., Barnett, J., Brownlow, A., Davison, N.J., Deaville, R., Galloway, T.S., Lindeque, P.K., Santillo, D., Godley, B.J., 2019. Microplastics in marine mammals stranded around the British coast: ubiquitous but transitory? Sci. Rep. 9. https://doi.org/10.1038/s41598-018-37428-3

Novillo, O., Raga, J.A., Tomás, J., 2020. Evaluating the presence of microplastics in striped dolphins (Stenella coeruleoalba) stranded in the Western Mediterranean Sea. Mar. Pollut. Bull. 160, 111557. https://doi.org/10.1016/j.marpolbul.2020.111557

Pham, C.K., Rodríguez, Y., Dauphin, A., Carriço, R., Frias, J.P.G.L., Vandeperre, F., Otero, V., Santos, M.R., Martins, H.R., Bolten, A.B., Bjorndal, K.A., 2017. Plastic ingestion in oceanic-stage loggerhead sea turtles (Caretta caretta) off the North Atlantic subtropical gyre. Mar. Pollut. Bull. 121, 222–229. https://doi.org/10.1016/j.marpolbul.2017.06.008

Philipp, C., Unger, B., Ehlers, S.M., Koop, J.H.E., Siebert, U., 2021. First Evidence of Retrospective Findings of Microplastics in Harbour Porpoises (Phocoena phocoena) From German Waters. Front. Mar. Sci. 8. https://doi.org/10.3389/fmars.2021.682532

Ryan, P.G., Cole, G., Spiby, K., Nel, R., Osborne, A., Perold, V., 2016. Impacts of plastic ingestion on post-hatchling loggerhead turtles off South Africa. Mar. Pollut. Bull. 107, 155–160. https://doi.org/10.1016/j.marpolbul.2016.04.005

van Franeker, J.A., Bravo Rebolledo, E.L., Hesse, E., IJsseldijk, L.L., Kühn, S., Leopold, M., Mielke, L., 2018. Plastic ingestion by harbour porpoises Phocoena phocoena in the Netherlands: Establishing a standardised method. Ambio 47, 387–397. https://doi.org/10.1007/s13280-017-1002-y

Zhang, X., Luo, D., Yu, R.Q., Xie, Z., He, L., Wu, Y., 2021. Microplastics in the endangered Indo-Pacific humpback dolphins (Sousa chinensis) from the Pearl River Estuary, China. Environ. Pollut. 270, 116057. https://doi.org/10.1016/j.envpol.2020.116057

Zhu, J., Yu, X., Zhang, Q., Li, Y., Tan, S., Li, D., Yang, Z., Wang, J., 2019. Cetaceans and microplastics : First report of microplastic ingestion by a coastal delphinid , Sousa chinensis. Sci. Total Environ. 659, 649–654. https://doi.org/10.1016/j.scitotenv.2018.12.389
